# Slaying the Serpent: Pearls and Pitfalls of Crotalid Envenomations

**DOI:** 10.7759/cureus.96847

**Published:** 2025-11-14

**Authors:** Nikolas P Foresteire, Tyler Kellett, Krishen Gosine, Kelly Grabbe, Anthony Jackson, Cory Howard

**Affiliations:** 1 Emergency Medicine Residency Program, Hospital Corporation of America (HCA) Florida, University of South Florida, Morsani College of Medicine Graduate Medical Education Consortium at Brandon Hospital, Brandon, USA

**Keywords:** crotalid, emergency medicine, snake bite, toxicology, toxicology and envenomation

## Abstract

Crotalid envenomations pose a significant clinical challenge for emergency medicine physicians, largely due to the potential for severe local toxicity, extensive tissue damage, and even systemic toxicity. This case report reviews the clinical presentation and anti-venom management of a patient who presented to our local emergency department after sustaining a crotalid envenomation to the left hand. Our case highlights common pitfalls in emergency management, including the non-interchangeability of dosing for available North American antivenoms. We briefly discuss the comparative efficacy of the two available North American antivenoms, CroFab® (BTG International, Inc., West Conshohocken, PA) and Anavip® (Rare Disease Therapeutics, Inc., Poway, CA).

## Introduction

Crotalid envenomation, particularly from the *Crotalus adamanteus* (Eastern Diamondback Rattlesnake), presents a complex medical challenge for clinicians due to antivenom supply, severe local tissue damage, systemic toxicity, and the possibility of venom-induced consumptive coagulopathy (VICC) [1]. VICC is a controversial term characterized by fibrinogen depletion, thrombocytopenia, and prolonged clotting times, necessitating timely intervention with appropriate antivenom therapy to mitigate life-threatening complications [2].

In North America, two primary antivenoms are available for the treatment of crotalid envenomation. They include Crotalidae polyvalent immune Fab (ovine) (CroFab®, BTG International, Inc., West Conshohocken, PA) and Crotalidae immune F(ab')₂ (equine) (Anavip®, Rare Disease Therapeutics, Inc., Poway, CA). Each has similar, yet distinct pharmacokinetic properties and clinical implications, particularly regarding dosing strategies. Ensuring optimal patient outcomes requires early consultation with regional poison control centers, which are critical resources for guiding evidence-based antivenom acquisition and administration, laboratory monitoring, and supportive care.

This case report details a severe Eastern Diamondback Rattlesnake envenomation complicated by VICC that presented to our local community emergency department, requiring coordinated multidisciplinary management. Our case was complicated by anti-venom dosing and administration, originally thought to be interchangeable between two different available products. We highlight the role of toxicologists, intensivists, and pharmacists in facilitating appropriate transfer, antivenom selection, and ongoing coagulation monitoring. This case underscores the importance of an integrated approach to envenomation management to optimize patient safety and therapeutic outcomes while minimizing common dose-related mistakes.

This case report was submitted, accepted, and presented as an outline at HCA Florida Brandon Hospital Research Day on April 17th, 2025.

## Case presentation

**Figure 1:**
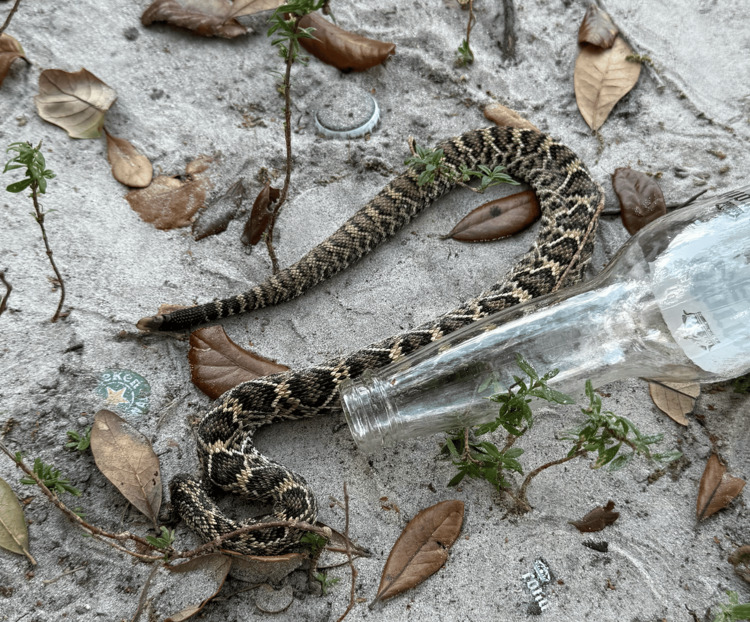
North American crotalid. Snake identification photo taken by the emergency medical services (EMS) crew before transport. The crotalid above was successfully identified as an Eastern Diamondback Rattlesnake.

A 52-year-old male presented to the emergency department after sustaining a rattlesnake envenomation. He was reaching down to pick up an item in his yard when he sustained two separate bites to the dorsum of the left hand with immediate pain. Local emergency medical services (EMS) were able to capture a photo of the snake for identification prior to transport (Figure 1). En route to the emergency department, he developed worsening swelling of his left hand with systemic signs including nausea, multiple episodes of non-bloody emesis, and diffuse abdominal pain. He denied any other symptoms such as difficulty breathing, drooling, confusion, headaches, numbness, or tingling. The patient's vital signs upon arrival are listed in Table 1.

**Table 1:** Initial presenting vital signs and repeat vital signs before transfer.

Vital signs	Arrival	Transfer (98 minutes later)
Mean arterial pressure (MAP)	93	98
Blood pressure	138/66	128/77
Heart rate	87	88
Oxygen saturation	100%	97%
Oxygen delivery	Room air	Room air
Temperature	37.1°C	36.9°C

Physical examination

**Figure 2:**
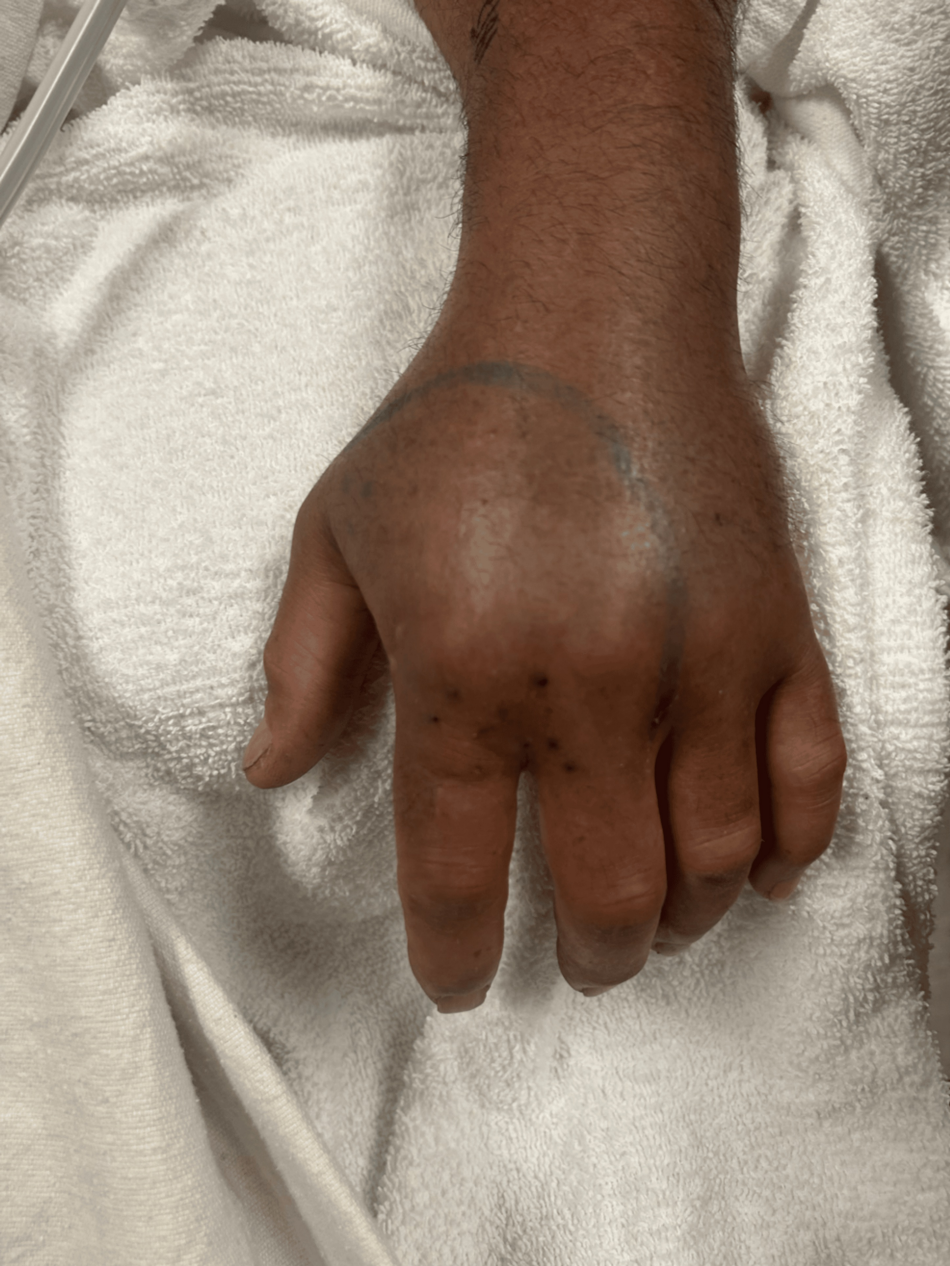
Dorsum of the left hand, noting four puncture wounds sustained from an Eastern Diamondback Rattlesnake. Initial demarcation of swelling and erythema performed by bedside clinician with surgical marker.

There were four puncture wounds to the dorsum of the left hand overlying the metacarpophalangeal joint of the second digit, with diffuse swelling and erythema to the hand as seen in Figure 2. There was diffuse tenderness. Compartments were tense. Distal sensation was intact. Brisk capillary refill was present in all digits. Radial pulse 2+. No active hemorrhaging, bullae, necrosis, or subcutaneous emphysema were visualized.

Clinical management

**Figure 3:**
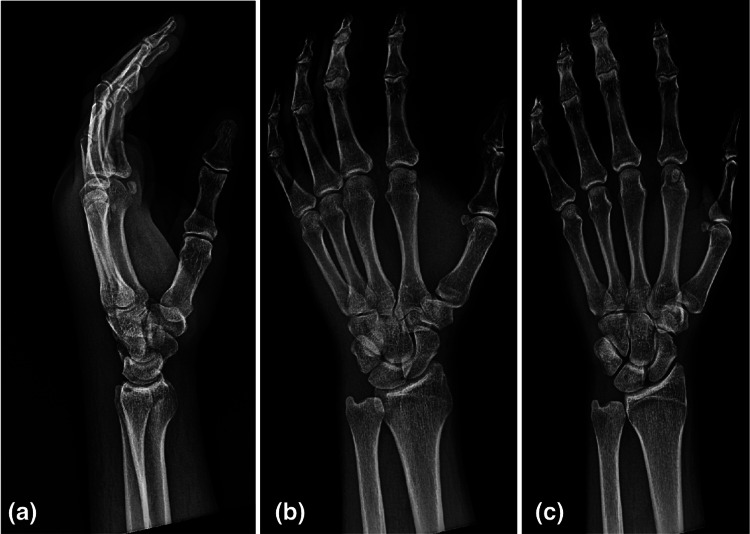
X-rays of the left hand with no osseous abnormalities or foreign bodies identified. (a) Notes a lateral X-ray of the left hand. (b) Oblique view of the left hand. (c) Posterior-anterior view of the left hand.

**Figure 4:**
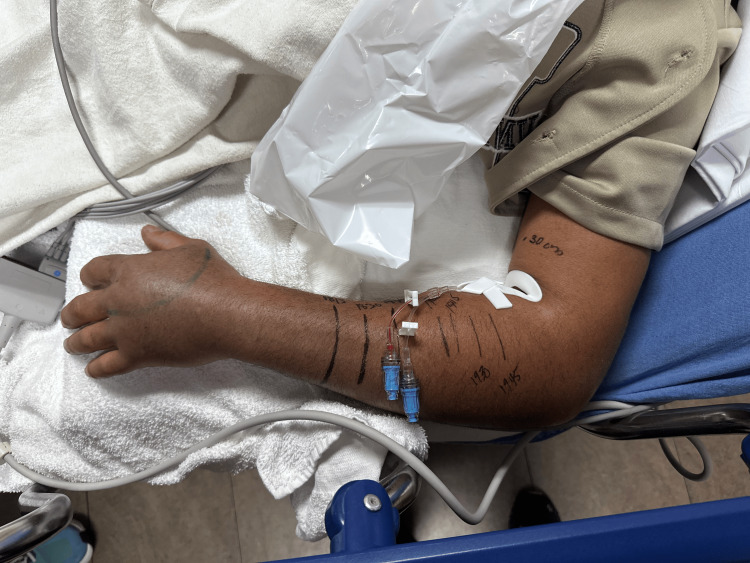
Circumferential measurements were taken upon presentation and upon transport to a tertiary care facility. The dorsum of the left hand was demarcated for swelling, erythema, and ecchymosis upon presentation, as noted by the curved green line.

**Figure 5:**
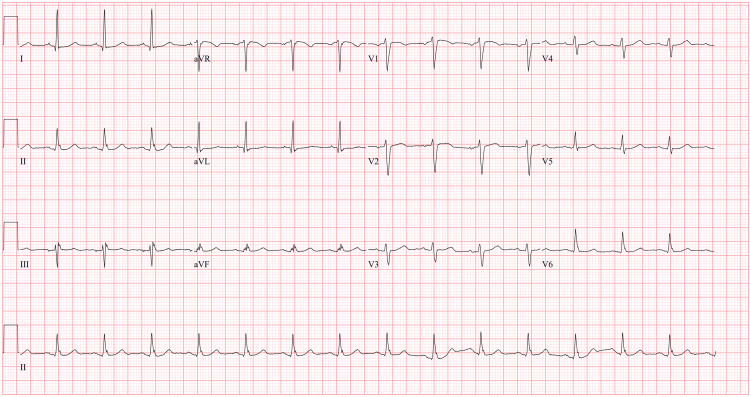
Electrocardiogram (ECG) performed, noting a normal sinus rhythm at a rate of 85 beats per minute. No acute ischemic changes or QT prolongation noted.

After obtaining intravenous access, the patient was administered 4 mg of intravenous ondansetron, 4 mg of intravenous morphine, and updated tetanus immunization via intramuscular injection with tetanus toxoid/reduced diphtheria toxoid/acellular pertussis vaccine adsorbed. Laboratory results and imaging were obtained as noted in Tables 2-4. X-ray imaging of the left hand was obtained to ensure there was no evidence of subcutaneous gas formation, retained foreign bodies, or bony abnormalities (Figure 3). Clinical re-evaluation of swelling and compartment sizes, including circumferential measurements, was taken of the left wrist, forearm, elbow, and upper arm every 15 minutes (Figure 4). An electrocardiogram (ECG) was performed with no signs of myocardial injury to suggest coronary envenomation, QT prolongation, or concerning electrolyte abnormalities before medication administration (Figure 5).

**Table 2:** Complete blood count obtained upon initial presentation and prior to tertiary facility transport. MCV: mean corpuscular volume, MCH: mean corpuscular hemoglobin, MCHC: mean corpuscular hemoglobin concentration, RDW: red cell distribution width, MPV: mean platelet volume, Hgb: hemoglobin, Hct: hematocrit.

	Arrival	Transfer (98 minutes later)
WBC	16 (H)	20 (H)
RBC	5.03	5.36
Hgb	14.8	15.8
Hct	45.1	47.1
MCV	89.7	87.9
MCH	29.4	29.5
MCHC	32.8	33.5
RDW	11.3 (L)	12.1
Platelets	26 (L)	14 (CL)
MPV	10.5	10.6
Platelet morphology	Marked decrease	Marked decrease

**Table 3:** Coagulation studies obtained upon initial presentation and prior to tertiary facility transport. PT: prothrombin time, INR: international normalized ratio, aPTT: activated partial thromboplastin time.

	Arrival	Transfer (98 minutes later)
PT	11.1	14.2 (H)
INR	1.0	1.3 (H)
aPTT	28	28
Fibrinogen	194 (L)	59 (L)

**Table 4:** Complete metabolic panel obtained upon initial presentation and prior to tertiary facility transport. BUN: blood urea nitrogen, ALT: alanine transaminase, AST: aspartate aminotransferase, GFR: glomerular filtration rate.

	Arrival	Transfer (98 minutes later)
Sodium	140	141
Potassium	3.7	3.6
Chloride	104	104
Carbon dioxide	23.1	24.4
Anion gap	12.9	12.6
BUN	9	10
Creatinine	0.82	0.82
Est GFR	>90	>90
BUN/creatinine ratio	10	12
Glucose	98	113 (H)
Calcium	9.9	9.7
Phosphorus	2.9	N/A
Magnesium	1.9	N/A
Total bilirubin	0.4	0.8
AST	29	31
ALT	29	30
Alkaline phosphatase	89	86
Creatinine kinase	124	N/A
Total protein	7.5	7.4
Albumin	4.9 (H)	4.7
Globulin	2.6	2.7
Albumin/globulin ratio	1.9 (H)	1.7

Due to signs of disseminated intravascular coagulation with rapidly expanding swelling, the poison control center recommended administration of antivenom once crossing a joint space. The pharmacy was consulted for the initial dosing of Crotalidae polyvalent immune Fab as recommended by the poison control center. Dosing was adjusted by the central pharmacist and sent to the bedside for administration of six vials. It was later recognized that Crotalidae immune F(ab')₂ was prepared by the pharmacy at an inadequate loading dose. Our facility only carried Crotalidae immune F(ab')₂; however, dosing was based on protocol from Crotalidae polyvalent immune Fab. After recognition, the patient was given another four vials to adhere to the proper Anavip® loading dose and ensure efficacy and patient safety, as described later in the discussion section. The intensive care unit (ICU), plastic surgery, and internal medicine (IM) teams were consulted by the emergency medicine team for multi-modal care and observation. Through contact with the medical toxicology at the local poison control center, pharmacy, IM, and plastic surgery, it was determined that the patient was displaying concerning clinical signs of consumptive coagulopathy with rapidly worsening swelling. Attempts were made to obtain further Anavip® at nearby facilities. After review of the available stock, it was determined that if the patient required further control, antivenom would not be readily available promptly. The need for a higher level of care with expanded storage of antivenom was deemed necessary by all consulting specialists and recommended by medical toxicology. The patient was transported via helicopter to a nearby tertiary care facility for further antivenom treatment and supportive care. 

Outcome

Upon arrival at the tertiary care facility, the patient was successfully transferred and observed without any repeat dosing of antivenom. Per consultation, his platelet count, INR, and fibrinogen began to increase and stabilize. Compartments were measured, and swelling decreased after the initial antivenom was administered before transport. The patient was discharged from the hospital after medical treatment with recommended outpatient follow-up. The patient was subsequently lost to follow-up.

## Discussion

Defined as belonging to the Crotalidae (pit viper or Viperidae) family, these serpents are named due to heat-sensing pits between their eyes and nostrils [3]. Eastern Diamondback Rattlesnakes (*Crotalus adamanteus*) are found across all regions of Florida. Other crotalids include the Dusky Pygmy Rattlesnake (*Sistrurus miliarius barbouri*), Florida Cottonmouth (*Agkistrodon conanti*), Eastern Copperhead (*Agkistrodon contortrix*), and Canebreak “Timber” Rattlesnake (*Crotalus horridus*) [4,5]. 

Crotalid envenomations account for over 10,000 emergency department (ED) visits in the United States annually [6]. Crotalids experience an annual peak in envenomations as the typical mating season occurs for most species between April and October (spring to late summer/fall), with a gestational period of approximately three to five months. Most offspring are born from July to October [7-9]. These periods represent when patients are at the highest risk of sustaining a bite. Presenting shortly after envenomation, outcomes are highly dependent on physician-led, timely intervention with one of the two available antivenoms when clinically appropriate.

The management of Crotalid envenomations presents several challenges, particularly regarding antivenom selection, dosing strategies, and healthcare system limitations. One of the most frequent pitfalls is the misconception that Crotalidae polyvalent immune Fab (CroFab®) and Crotalidae immune F(ab')2 (Anavip®) are interchangeable, leading to dosing errors, treatment delays, and suboptimal patient outcomes, including lack of symptomatic control or progression of envenomation. These two antivenoms differ in their pharmacokinetics, mechanism of action, and dosing requirements, which must be carefully considered when selecting an appropriate treatment regimen.

Indications for antivenom administration

Antivenom should be considered in any patient showing signs of systemic toxicity or severe local effects, as these can rapidly progress and require urgent intervention in conjunction with a local medical toxicologist. Indications for antivenom administration include: (1) Necrosis or severe swelling beyond a major joint space. (2) Coagulation abnormalities, including platelet count <150, prothrombin time (PT) >15 seconds, or Fibrinogen <150. (3) Systemic effects including hemodynamic instability, angioedema, or acute encephalopathy [10].

Available formulations in the United States

Two main types of antivenom are used for the treatment of pit viper envenomations: Crotalidae polyvalent immune Fab (CroFab®) and Crotalidae immune F(ab')2 (Anavip®). Both antivenoms are FDA-approved and can be used for North American rattlesnake, copperhead, and cottonmouth envenomations, with key differences.

*Crotalidae Polyvalent Immune Fab (CroFab*®*)*

CroFab® is an ovine-derived (sheep) antivenom, formulated as a single Fab fragment. It is FDA-approved for adults and children. It is indicated for rattlesnakes, copperheads, and cottonmouths (*Crotalus atrox*, *Crotalus adamanteus*, *Crotalus scutulatus*, *Agkistrodon piscivorus*) [10]. CroFab® consists of papain-digested, ovine-immunized Fab fragments that bind to and neutralize Crotalid venom components, facilitating renal elimination. Due to its small molecular size, CroFab® exhibits rapid distribution into the extracellular space, allowing for effective initial venom neutralization but also contributing to a short serum half-life of approximately 12-23 hours. As a result, venom effects may recur after initial control, necessitating scheduled maintenance dosing to sustain therapeutic levels [10,11]. Initial dosing includes administration of four to six vials intravenously over one hour, followed by additional dosing as necessary. The total number of intravenous vials may be up to six vials over 18 hours, depending on patient response. Reported adverse effects include serum sickness (fever, arthralgia, myalgias), recurrent coagulopathy, and concerning allergic reactions like anaphylaxis or angioedema. Administration poses challenges, including required careful reconstitution (18 mL of saline per vial) and slow “pill-roll” mixing. If shaken, denaturation of the antivenom may occur. The entire dose must be further diluted, and the vials must be stored in a refrigerator [11].

*Crotalidae Immune F(ab*'*)2 (Anavip*®​​​​​​​*)*

Anavip®, derived from equine (horse) serum, uses a double Fab fragment that targets venom toxins more efficiently [10,11]. It is currently FDA-approved for use in adults, pregnancy, and children. It is indicated for all North American pit viper species (*Bothrops asper*, *Crotalus simus*, *Crotalus adamanteus*, *Crotalus atrox*, *Crotalus scutulatus*, *Agkistrodon contortrix*, *Agkistrodon piscivorus*) [11]. Anavip® is composed of equine-immunized, intact F(ab’)2 fragments, which are larger than Fab fragments and lack the Fc portion of IgG, reducing immunogenicity. These larger antibody fragments have a longer serum half-life of approximately 133 hours, providing prolonged venom neutralization and potentially reducing the risk of recurrent toxicity. Due to its extended systemic presence, Anavip® does not require scheduled maintenance dosing, offering a distinct advantage in preventing late coagulopathy or recurrent swelling. Adverse effects include pruritus, nausea, headache, and myalgia have been reported. Notably, it has a lower reported incidence of recurrent coagulopathy and requires fewer repeat doses. Anavip® has two binding sites per fragment, which enhances venom neutralization [11]. It can be reconstituted in seconds, stored at room temperature, and has a shelf life reported up to three years. While having potential benefits, availability may be the largest issue, as many hospitals may not stock Anavip® [10,11].

Dosing-related errors

This case highlights a critical error in envenomation management that arises from dosing-related mistakes, particularly the incorrect assumption that CroFab® and Anavip® share identical dosing protocols. These differences must be carefully understood to avoid underdosing, delayed symptom control, and increased hospital length of stay. The initial dose of Crotalidae polyvalent immune Fab is four to six vials, followed by reassessment at one-hour intervals. If initial control is not achieved, an additional four to six vials may be administered. Once control of coagulopathy and swelling is established, maintenance dosing of two vials every six hours for three doses is recommended to reduce the risk of recurrence [10]. In contrast, the standard regimen for Crotalidae immune F(ab')2 consists of a fixed 10-vial dose, with repeat doses as needed for ongoing symptoms. Unlike Crotalidae polyvalent immune Fab, Crotalidae immune F(ab')2 does not require scheduled maintenance dosing, as its extended half-life provides sustained venom neutralization.

Failure to recognize these differences may result in pharmacists or providers mistakenly substituting maintenance doses of CroFab® with Anavip®, leading to unnecessary treatment or underutilization of Anavip®'s longer-lasting effects. Additionally, delays in treatment initiation may occur if pharmacy staff are unaware of formulary limitations or incorrectly assume both antivenoms require identical reconstitution and administration times [11]. Delays in antivenom administration due to confusion over dosing, hospital formulary restrictions, or insurance concerns can lead to progressive local tissue damage, coagulopathy, and systemic toxicity. Some hospitals may only stock one antivenom, limiting treatment flexibility. Institutions carrying only CroFab® may encounter difficulties in patients requiring prolonged therapy, while those stocking only Anavip® may need to ensure providers understand its different pharmacokinetic properties and lack of maintenance dosing requirements.

This case highlights the importance of pharmacy education, interdisciplinary communication, and standardized treatment protocols to prevent errors in antivenom selection and dosing. Emergency physicians, toxicologists, pharmacists, and critical care teams must be well-versed in the distinct properties of CroFab® and Anavip® to optimize patient outcomes. Implementing hospital-specific guidelines, education initiatives, and decision-support tools can help reduce errors, minimize delays, and improve overall envenomation management. Effective management of crotalid envenomation involves collaboration with an interdisciplinary team. In this case, following the initial assessment and stabilization in the emergency department (ED), the patient's care was coordinated with specialists in toxicology, poison control, and critical care. Early contact with poison control (1-800-222-1222) is essential for expert guidance on antivenom dosing and further management. After administration of the first dose of Anavip®, the patient required transfer to a tertiary care center with an intensive care unit (ICU) for further monitoring, given the severity of the envenomation. The transfer was carefully coordinated to ensure continuous care, and the patient was monitored for potential late effects, including delayed coagulopathy and systemic complications.

Other common pitfalls

Prophylactic antibiotics are not routinely recommended and should only be given for secondary infections. Other recommendations include the avoidance of a tourniquet, application of ice, deroofing of wounds, or fasciotomies. Compartment swelling and pressures usually improve significantly with antivenom administration.

## Conclusions

The management of pit viper envenomations demands a swift and systematic approach, emphasizing rapid assessment, prompt antivenom administration, and vigilant monitoring. Both CroFab® and Anavip® serve as effective treatment options, but are often limited by the regional or hospital availability. Familiarity with initial dosing differences should be protocolized by institutions to ensure patients are receiving adequate dosing. Optimal patient outcomes hinge on the early involvement of an interdisciplinary team, including toxicologists, poison control specialists, and critical care providers, ensuring comprehensive care and timely interventions.

This case underscores the importance of timely antivenom administration, meticulous monitoring, and transfer to a tertiary care center, which collectively contributed to a successful patient outcome. Additionally, it highlights key challenges clinicians face, including antivenom selection, dosing strategies, and accessibility. It also reinforces the need for a protocolized and informed approach to envenomation management by bedside clinicians. Take-home points may include: Institutional protocol initiation is recommended for crotalid envenomations to improve the efficacy of care; Familiarity with the indications and dosing for crotalid antivenom administration is vital; Supply chain recognition for individual institutions is highly encouraged to determine options during shortages or demand; Antivenom dosing is not interchangeable. Initial CroFab® dosing is four to six vials, while Anavip® is 10 vials.

## References

[1] Maduwage K, Isbister GK (2014). Current treatment for venom-induced consumption coagulopathy resulting from snakebite. PLoS Negl Trop Dis.

[2] Isbister GK (2010). Snakebite doesn't cause disseminated intravascular coagulation: coagulopathy and thrombotic microangiopathy in snake envenoming. Semin Thromb Hemost.

[3] Greene S, Cheng D, Vilke GM, Winkler G (2025). How should native crotalid envenomation be managed in the emergency department?. J Emerg Med.

[4] Ernst CH, Ernst EM (2003). Snakes of the United States and Canada. https://www.researchgate.net/publication/291808555_Snakes_of_the_United_States_and_Canada_by_Carl_H_Ernst_and_Evelyn_M_Ernst_2003_Smithsonian_Books_Washington.

[5] Krysko KL, Enge KM, Moler PE (2019). Amphibians and Reptiles of Florida.

[6] Sheikh S, Leffers P (2018). Emergency department management of North American snake envenomations. Emerg Med Pract.

[7] Aldridge RD, Duvall D (2002). Evolution of the mating season in the pitvipers of North America. Herpetol Monogr.

[8] Taylor EN, DeNardo DF, Jennings DH (2004). Seasonal steroid hormone levels and their relation to reproduction in the Western Diamond-backed Rattlesnake, Crotalus atrox (Serpentes: Viperidae). Gen Comp Endocrinol.

[9] (2025). U.S. Food and Drug Administration. CroFab (Crotalidae polyvalent immune Fab (ovine)) prescribing information. https://www.fda.gov/media/74683/download.

[10] Buchanan JT, Thurman J (2025). Crotalidae envenomation. StatPearls (Internet).

[11] (2025). U.S. Food and Drug Administration. ANAVIP (Crotalidae immune F(ab’)2 (equine)) prescribing information. https://www.fda.gov/media/92139/download.

